# Observation on the efficacy of different methylprednisolone regimens in the treatment of myasthenia gravis

**DOI:** 10.12669/pjms.38.4.5069

**Published:** 2022

**Authors:** Xiaoting Lin, Guoyan Qi

**Affiliations:** 1 Xiaoting Lin, Center of Treatment of Myasthenia Gravis Hebei Province, Shijiazhuang People’s Hospital, Shijiazhuang, 050000, Hebei, China; 2 Guoyan Qi, Center of Treatment of Myasthenia Gravis Hebei Province, Shijiazhuang People’s Hospital, Shijiazhuang, 050000, Hebei, China

**Keywords:** Lymphocyte subsets, Methylprednisolone, Myasthenia gravis, Periodic therapy, Pulse therapy

## Abstract

**Objective:**

To investigate the efficacy of different methylprednisolone regimens in the treatment of myasthenia gravis (MG).

**Methods:**

A total of 98 patients with MG admitted to Shijiazhuang People’s Hospital from December 2018 to November 2019 were randomly divided into two groups, with 49 cases in each group. Patients in the control group received high-dose methylprednisolone pulse therapy, while those in the experimental group received medium-dose periodic therapy of methylprednisolone. Anti-acetylcholine receptor antibodies (AChRab), clinical absolute scores, complement levels (C3, C4), T lymphocyte subsets (CD3+, CD4+, CD4+CD25+), cytokines [interferon-γ (INF-γ), transforming growth factor-β1 (TGF-β1), interleukin-6 (IL- 6), interleukin-18 (IL-18)], and changes in quality of life [15-item Myasthenia Gravis Quality of Life Scale (MGQOL-15) score] were compared between the two groups before treatment and one and three months after treatment. Moreover, the incidence of adverse drug reactions in the two groups during 3 months of treatment was compared.

**Results:**

After three months of treatment, ACHRAB, clinical absolute score, CD3+, CD4+, INF-γ, IL-6, IL-18 and MGQOL-15 scores in both groups were significantly decreased compared with those before treatment (p<0.05), and the scores of C3, C4, CD4+CD25+ and TGF-β1 in both groups were significantly higher than those before treatment (p<0.05), and the experimental group had more significant changes than the control group (p<0.05). During three months of treatment, the total incidence of adverse drug reactions in the experimental group was significantly lower than that in the control group (p<0.05).

**Conclusions:**

The medium-dose periodic therapy of methylprednisolone is more prominent in the long-term efficacy performance. It can improve the immunity and quality of life of patients, and it is safer and has high clinical application value.

## INTRODUCTION

Myasthenia gravis (MG) is an acquired autoimmune disease mediated by acetylcholine receptor antibody (AChRab), dependent on cellular immunity and involved in complement. It mainly involves acetylcholine receptors on the postsynaptic membrane at the neuromuscular junction (NMJ).^1^-^3^ Patients suffering from MG are mainly characterized by the fluctuation of certain specific striated muscle groups and the symptoms of muscle weakness prone to fatigue.^4^,^5^ Glucocorticoids are currently the preferred treatment regimen for adults with MG^6^,^7^, while conventional treatment includes high-dose methylprednisolone pulse therapy and small-dose escalation regimen. With a high-dose methylprednisolone pulse therapy, significant efficacy can be achieved, but transient myasthenia may be aggravated within a short period of time after treatment, resulting in an increased risk of inducing myasthenia gravis crisis and more adverse reactions. With a small-dose escalation regimen, exacerbation of the disease may still be caused, and the treatment response of patients is often difficult to predict, so it is not recommended as the mainstream treatment regimen.^8^-^10^ In view of this, effective hormone therapy with few side effects is urgently needed to be found. In this study, the medium-dose periodic therapy of methylprednisolone adopted by our hospital in recent years was introduced into the treatment of MG, so as to investigate the difference in efficacy between this regimen and high-dose methylprednisolone pulse therapy. The results obtained are now reported as follows.

## METHODS

A total of 98 patients with MG admitted to Shijiazhuang People’s Hospital from December 2018 to November 2019 were selected as subjects and divided into two groups according to random number table: the experimental group and the control group, with 49 cases in each group.

### Ethical approval:

The study was approved by the Institutional Ethics Committee of Shijiazhuang People’s Hospital at June 10, 2021(No.93), and written informed consent was obtained from all participants

### Case inclusion criteria:

- Patients who meet the relevant diagnostic criteria in MG;^11^
- Patients aged between 18 and 70 years;
- Patients who gave informed consent to this study.
- Case exclusion criteria:
- Patients who received high-dose hormone therapy within 3 months prior to this study;
- Patients who were treated with gamma globulin and plasma exchange within the first 3 months of this study;
- Patients with pneumonia, respiratory failure and thymus tumors;
- Patients with dysfunction of vital organs such as liver and kidney.

In the experimental group, there were 18 males and 31 females, aged 19-62 years old, with an average of (41.36±8.60) years old. Osseman typing: 26 cases of Type-IA, 13 cases of Type-IIA and 10 cases of Type-IIB. In the control group, there were 16 males and 33 females, aged 21-63 years old, with an average age of (41.79±8.04) years; Osseman typing: 24 cases of Type-IA, 15 cases of Type IIA, and 10 cases of Type-IIB. No statistical difference can be observed in the general information of the two groups (p>0.05), which were comparable.

Patients in the two groups were takeing orally 2 mg/(kg•d) immunosuppressant azathioprine (Beijing Jialin Pharmaceutical Co., Ltd., State Drug Approval No.: H20003841, 100mg*18 tablets*2 tablets). The control group was given a high-dose methylprednisolone pulse therapy (Sinopharm Group Rongsheng Pharmaceutical Co, Ltd., State Drug Approval No.: H20040844, 0.25g injection) with intravenous drip of 1000 mg/d, and the dosage was adjusted to 500 mg/d, 250 mg/d, 125 mg/d, 60 mg/d and finally 40 mg/d after every three consecutive days of treatment for 3 months. In contrast, the experimental group was given a medium-dose periodic therapy of methylprednisolone, i.e., intravenous injection of 7 mg/(kg.d) for five days, with three weeks as a cycle, lasting for 1-2 cycles.

### Evaluation criteria:

Absolute clinical MG score includes 8 items, including upper eyelid muscle strength, upper eyelid fatigue test, level of eye movement, facial muscle strength, respiratory function, swallowing function, upper limb fatigue test, and lower limb fatigue test^12^, with different scores for each item, with a total score of 0-60. The higher the score, the more severe the MG symptoms of patients. The 15-item Myasthenia Gravis Quality of Life Scale (MGQOL-15) includes mobility, symptoms, mental health, overall satisfaction and other areas^13^, with a total of 15 items, 0-4 points for each item, and 0-60 points in total. The higher the score, the worse the quality of life of patients.

Five mililiter of peripheral venous blood was collected before treatment, 1 month and 3 months after treatment. After static centrifugation, the serum was separated, and the levels of anti-acetylcholine receptor antibody (AChRab) were determined by radioimmunoassay. The levels of interferon-γ (INF-γ), transforming growth factor-β1 (TGF-β1) and interleukin-6 (IL-6) were determined by enzyme-linked immunosorbent assay (ELISA). Moreover, the levels of complement C3 and C4 were determined by immunoscattering turbidimetry, CD3+, CD4+, CD4+CD25+ were determined by flow cytometry, and interleukin-18 (IL-18) was determined by the double antibody sandwich method.

### Observation indexes:

AChRab, clinical absolute score, complement levels (C3, C4), T lymphocyte subsets (CD3+, CD4+, CD4+CD25+), cytokines (INF-γ, TGF-β1, IL- 6, IL-18), and changes in quality of life (MGQOL-15 score) were compared between the two groups before treatment and one month and three months after treatment. Moreover, the incidence of adverse drug reactions in the two groups during 3 months of treatment was compared.

### Statistical methods:

All the data were statistically analyzed by SPSS21.0 software, and the measurement data were expressed as (*x*±*s*). Two independent sample t-test was used for inter-group data analysis, and paired t test was used for intra-group comparison during the same period. Furthermore, enumeration data were expressed as number of cases and percentage (%), and χ2 test was used for comparison between groups. P<0.05 indicates a statistically significant difference.

## RESULTS

After three months of treatment, ACHRAB and clinical absolute score in both groups were significantly decreased compared with those before treatment (p<0.05), and the scores of C3 and C4 in both groups were significantly higher than those before treatment (p<0.05), and the experimental group had more significant changes than the control group(p<0.05), as shown in Table-I.

**Table-I:** Comparison of ACHRAB, absolute clinical score and levels of C3 and C4 between the two groups (x±s).

Group	Time	No. of cases	AChRab (nmol/L)	Clinical absolute score (points)	C3 (g/L)	C4 (g/L)
Experimental group	Before treatment	49	0.89±0.07	25.89±3.64	0.99±0.12	0.18±0.03
	1 month after treatment	49	0.61±0.06*	18.20±2.36*	1.15±0.17*	0.22±0.03*
	3 months after treatment	49	0.41±0.05*^#^	11.56±2.41*^#^	1.42±0.18*^#^	0.29±0.04*^#^
Control group	Before treatment	49	0.88±0.08	26.03±3.78	1.01±0.10	0.19±0.03
	1 month after treatment	49	0.56±0.07*	17.56±2.15*	1.18±0.16*	0.23±0.03*
	3 months after treatment	49	0.48±0.07*	13.86±2.05*	1.32±0.15*	0.25±0.03*

***Note:***

* p<0.05 compared with that before treatment in this group;

# p<0.05 compared with that in the control group at the same time.

After three months of treatment, the levels of CD3+ and CD4+ in both groups were significantly decreased compared with those before treatment (p<0.05), while the score of CD4+CD25+ in both groups was significantly higher than that before treatment (p<0.05), and the experimental group had more significant changes than the control group(p<0.05), as shown in Table-II.

**Table-II:** Comparison of the levels of CD3+, CD4+ and CD4+CD25+ between the two groups (x±s, %).

Group	Time	Number of cases	CD3+	CD4+	CD4+CD25+
Experimental group	Before treatment	49	75.35±8.94	46.85±6.42	17.84±2.17
	1 month after treatment	49	69.63±7.30*	40.25±4.04*	21.26±1.85*
	3 months after treatment	49	59.49±7.02*^#^	33.69±3.84*^#^	25.54±1.71*
Control group	Before treatment	49	75.09±9.20	46.81±6.59	17.89±2.06
	1 month after treatment	49	67.79±8.32*	39.68±4.10*	21.45±1.78*
	3 months after treatment	49	63.63±6.28*	35.96±3.98*	22.09±1.80*

***Note:***

* p<0.05 compared with that before treatment in this group;

# p<0.05 compared with that in the control group at the same time.

After three months of treatment, the levels of INF-γ, IL-6 and IL-18 in both groups were significantly lower than those before treatment (p<0.05), and TGF-β1 scores in both groups were significantly higher than those before treatment (p<0.05), and the experimental group had more significant changes than the control group(p<0.05), as shown in Table-III.

**Table-III:** Comparison of INF-γ, TGF-β1, IL-6 and IL-18 between the two groups (x±s).

Group	Time	No. of cases	INF-γ(pg/ml)	TGF-β1(ng/ml)	IL-6(pg/ml)	IL-18(pg/ml)
Experimental group	Before treatment	49	120.64±13.49	2.01±0.18	97.68±11.36	236.84±40.63
	1 month after treatment	49	72.63±10.31*	2.39±0.17*	48.69±7.29*	89.36±13.08*
	3 months after treatment	49	59.48±9.47*^#^	2.96±0.23*^#^	37.65±6.89*^#^	40.37±4.84*^#^
Control group	Before treatment	49	121.36±11.98	2.03±0.17	97.84±11.17	240.85±35.98
	1 month after treatment	49	70.69±10.85*	2.43±0.18*	46.86±8.30*	86.96±12.30*
	3 months after treatment	49	64.63±8.31*	2.80±0.18*	43.65±5.27*	55.36±6.85*

***Note:***

* p<0.05 compared with that before treatment in this group;

# p<0.05 compared with that in the control group at the same time.

After three months of treatment, the MGQOL-15 scores in both groups were significantly lower than those before treatment (p<0.05), and those in the experimental group was significantly lower than those in the control group (p<0.05), as shown in Table-IV. During three months of treatment, the total incidence of adverse drug reactions in the experimental group was significantly lower than that in the control group (p<0.05), as shown in Table-V.

**Table-IV:** Comparison of MGQOL-15 scores between the two groups (x±s, points).

Group	Time	No. of cases	Mobility	Symptoms	Mental health	Overall satisfaction
Experimental group	Before treatment	49	23.36±4.71	9.65±0.85	5.31±0.81	2.89±0.29
	1 month after treatment	49	19.63±2.21*	6.38±0.95*	3.62±0.59*	2.23±0.12*^#^
	3 months after treatment	49	10.63±2.48*^#^	3.65±0.54*^#^	2.82±0.33*^#^	1.76±0.12*^#^
Control group	Before treatment	49	23.41±4.51	9.46±0.79	5.27±0.79	2.85±0.28
	1 month after treatment	49	19.09±2.32*	6.45±0.82*	3.70±0.48*	2.27±0.14*
	3 months after treatment	49	13.28±1.96*	4.88±0.55*	3.52±0.39*	2.02±0.10*^#^

***Note:***

* p<0.05 compared with that before treatment in this group;

# p<0.05 compared with that in the control group at the same time.

**Table-V:** Comparison of the incidence of adverse drug reactions between the two groups [number of cases (%)].

Group	No. of cases	Electrolyte disturbance	Transient aggravation of muscle weakness	Peptic ulcer	Steroid diabetes	Total incidence
Experimental group	49	2(4.08)	3(6.12)	0(0.00)	1(2.04)	6(12.24)*
Control group	49	3(6.12)	17(34.69)	1(2.04)	1(2.04)	22(44.89)

***Note:***

* p<0.05 compared with the control group.

## DISCUSSION

MG is a type of autoimmune disease. Patients with MG may suffer from muscle group involvement in different parts owing to the conduction disorder at the neuromuscular junction, which can be manifested as skeletal muscle weakness, limb soreness and other symptoms. Cholinesterase inhibitors and immunosuppressive agents are currently the preferred therapeutic options for the treatment of the disease in clinical practice. However, the former is a temporary solution, which can relieve the symptoms of patients but cannot eliminate their symptoms^14^,^15^, and the efficacy of medication alone cannot last for a long time. The latter, represented by glucocorticoids, can improve the immune dysfunction of patients. Despite the remarkable efficacy of high-dose pulse therapy currently commonly used, its performance in adverse drug reactions is not optimistic, so further improvement of the medication regimen is needed to ensure medication safety.

AChRab is the primary pathogenic factor of MG. Due to the abnormally large width of the neuromuscular junction in patients with MG, the acetylcholine receptors are significantly reduced, and the deposition of Achrab becomes more serious, which significantly increases the serum expression level.^16^,^17^ On the other hand, complement also plays an important role in the pathogenesis of patients with MG. All kinds of complement components can eventually form a complement complex. With the deposition of complement complex on the cell surface, the biological activity of the cell may change due to its stimulation effect, affecting the disease performance of patients with MG^18^,^19^ In this study, after three months of treatment, the experimental group showed more significant changes in AChRab, clinical absolute score, C3 and C4 levels, suggesting that methylprednisolone high-dose pulse therapy and medium-dose periodic therapy of methylprednisolone had similar effects on the improvement of antibody, complement levels and symptom performance in the early treatment of patients with MG. In the long-term treatment, medium-dose periodic therapy boasts a more stable effect. The application of glucocorticoids can reduce the deposition of immune complexes by reducing complement consumption, thus showing an increase in the levels of complements C3 and C4. Despite the remarkable effect of high-dose pulse therapy in the early stage of treatment, the long-term efficacy of the treatment is not as good as that of the medium-dose periodic therapy as the dosage gradually decreases to the lowest effective dose in the later stage.

T lymphocyte subsets are the central link in the regulation of the immune function of the body and an important participant in the development of MG disease.^20^,^21^ It has been shown in current related studies that the levels of CD4+CD25+ can be significantly reduced in patients with unstable MG disease, leading to a significantly decreased inhibition of a large number of activated reactive T cells, thus exacerbating immune dysfunction and more prolonged disease. As cytokines secreted by Th1 and Th2 cells, the levels of INF-γ, IL-6 and IL-18 can reflect the immune balance maintained by Th1 and Th2 cells.^22^,^23^ In this study, after 3 months of treatment, the experimental group showed better performance in the above indexes, suggesting that high-dose methylprednisolone pulse therapy and medium-dose periodic therapy of methylprednisolone can effectively regulate the immune function and related cytokine secretion in patients with MG at the early stage of treatment, and promote the improvement of patients’ quality of life. In the long-term treatment, medium-dose periodic therapy boasts a more stable effect. In the early stage of high dose medication, about 40%-60% of the patients may experience the transient aggravation of muscle weakness and suffer from a high risk of other complications such as hypokalemia, which has a certain potential risk of medication safety. In this study, the total incidence of adverse drug reactions in the experimental group was significantly lower than that in the control group, which is similar to the above research background, suggesting that the medium-dose periodic therapy of methylprednisolone has higher drug safety while ensuring the efficacy.

### Limitations of this study:

The sample size of this study was small and the follow-up time was short. If the sample size can be further expanded, the conclusion may be more convincing. In addition, further studies with large-scale standard treatment are needed to verify the curative effect, safety, optimum dose, and duration of different methylprednisolone application regimens.

## CONCLUSION

Among different methylprednisolone application regimens, the efficacy of medium-dose periodic therapy of methylprednisolone in the early treatment of patients with MG is not significantly different from that of high-dose methylprednisolone pulse therapy. Medium-dose periodic therapy is more significant in terms of long-term efficacy, and boasts of better drug safety performance at the same time.

### Authors’ Contributions:

**XL** designed this study and prepared this manuscript, and are responsible and accountable for the accuracy or integrity of the work and significantly revised this manuscript.

**GQ** collected and analyzed clinical data.
